# Application of hyaluronic acid as carriers in drug delivery

**DOI:** 10.1080/10717544.2018.1450910

**Published:** 2018-03-14

**Authors:** Gangliang Huang, Hualiang Huang

**Affiliations:** a Active Carbohydrate Research Institute, Chongqing Normal University, Chongqing, P. R. China;; b School of Chemistry and Environmental Engineering, Wuhan Institute of Technology, Wuhan, P. R. China

**Keywords:** Hyaluronic acid, derivatives, drug carriers, drug delivery, tumor targeting

## Abstract

Hyaluronic acid has good biocompatibility, biodegradability, and nonimmunogenicity. In addition, it has the ability to recognize specific receptors that are overexpressed on the surface of tumor cells, and cancer drugs can be targeted to the tumor cells to better kill them. Therefore, hyaluronic acid has attracted much attention as drug delivery vehicle. Herein, the application of hyaluronic acid as carrier in drug delivery was analyzed and summarized in detail. It showed that hyaluronic acid would have broad prospects for drug delivery.

## 1. Introduction

Hyaluronic acid is a linear macromolecular mucopolysaccharide that is composed of alternatingly linked two saccharide units of glucuronic acid and *N*-acetylglucosamine (Alaniz et al., 2016). It has good biocompatibility, biodegradability, high viscoelasticity, and can be combined with specific receptor on the cell surface (Widjaja et al., 2014). Hyaluronic acid receptor CD44 has been found to be expressed at low level on the surface of epithelial, hematopoietic, and neuronal cells, but overexpressed in many tumor cells (Jong et al., 2012). In addition, some groups of hyaluronic acid such as hydroxyl, carboxyl, and *N*-acetyl are suitable for chemical modification. Therefore, hyaluronic acid and derivatives as drug carriers contribute to drug thickening, sustained release, transdermal absorption, and improve drug targeting.

Coupling of cytotoxic drug to macromolecular substance improves the pharmacokinetic profile of drug, prolongs drug distribution, and eliminating time (Zhang et al., 2014). In addition, the slow release of drug from the carrier allows the drug to remain in the tumor tissue at a higher concentration and lower plasma drug concentration. Hyaluronic acid and drug conjugates have been demonstrated to have the dual advantage of aggregation at the tumor site and receptor-mediated endocytosis (Ossipov, 2010). Hyaluronic acid and its derivatives have been widely used in various drug delivery systems, such as nanoparticle drug delivery system, gel drug delivery system, cationic polymer gene carrier system, nanoemulsion delivery system, polyelectrolyte microcapsule drug delivery system, microsphere drug delivery system, film delivery system, and so on. Some examples of applications are shown in Table 1. Based on the above analysis, the application of hyaluronic acid and its derivatives as drug carriers was analyzed and discussed in detail herein.

**Table 1.** The application of some hyaluronic acid delivery systems.

Dosing system type	Loaded drug	*In vivo* characteristics
Hyaluronic acid-methylcellulose hydrogel	α-Chymotrypsin, IgG	Slow release of drugs in 28 days
	Nimodipine	Submicron particles with slow release of drug within 2–3 days
Hyaluronic acid microsphere	Recombinant human insulin	Prolongation of retention time and half-life of the drug *in vivo*
Hyaluronic acid-aminoethyl *iso*-butylenate nanogel	Insulin, GLP-1, EPO	The drug release was affected by the cross-linking density and degradation rate of the gel, and had the sustained release characteristics
Thiolated hyaluronic acid microhydrogel	EPO	The stable release of drug in 7 days was maintained, and the blood concentration was higher than 0.1 μg/L

## 2. The application of hyaluronic acid and its derivatives as carriers in drug delivery

In view of the specific binding of hyaluronic acid to the receptors on the surface of cancer cells, its biodegradability and biocompatibility, the application of hyaluronic acid in the targeted drug delivery of anticancer drugs has made great progress. It can be used as a carrier and react with other drugs to form conjugates. The conjugates have the controlled release and targeted effect, which can target the delivery of multiple drugs to various pathological sites, so as to achieve the purpose of timing and directional release (Chen et al., 2014). However, hyaluronic acid is easily degraded in the human body (Bot et al., 2008). Therefore, a nitroxide-containing substance should be added to protect the hyaluronic acid from being degraded or a hyaluronidase inhibitor is added to prevent degradation of hyaluronic acid by inhibiting the activity of hyaluronidase (Sung et al., 2014).

### 2.1. Targeted cell delivery of nucleic acids

In recent years, cationic polymers have been extensively studied as gene delivery vehicles (Lv et al., 2006). Cationic polymers can improve the stability of DNA and penetrate cell membranes by the action of charges, escaping the degradation of endosomes. However, such gene vectors have the disadvantages of poor targeting, high cytotoxicity, and low transfection rate. Therefore, the study of targeted carrier materials to reduce cytotoxicity and increase the transfection rate has attracted much attention. Gene drug delivery system based on hyaluronic acid showed good advantages (Saravanakumar et al., 2014). Zhou et al. synthesized the amphipathic vector hyaluronic acid-PEI (HAP) for gene delivery by periodic acid oxidation of hyaluronic acid and PEI. This vector protected DNA from nuclease degradation well, isolated DNA from the complex, and was less toxic. HAP could overcome the shortcomings of PEI nonspecific transfection, and had high transfection rate in HepG2 cells, which could promote cell uptake more effectively (Yao et al., 2010). Hyaluronic acid-spermine conjugate was also synthesized from the structure of high-mobility group protein to improve the transfection efficiency of encapsulated DNA (Xu & Kc, 2016). Hyaluronic acid receptors are abundant in some specific tissues such as liver, kidney, and most tumor tissues (Rosso et al., 2013). Hyaluronic acid can bind to the receptor on the cell surface and be absorbed into the cell through the endocytosis mediated by the hyaluronic acid receptor. Synthetic hyaluronic acid-polylysine (PLL) conjugate targeted the HARE receptor in the sinusoidal epithelium of liver cells. They conjugated to induce the *ε*-amino group of hyaluronic acid-terminal PLL to synthesize a comb-type copolymer by reducing the amino group. The copolymer was used to form complex with DNA and was injected into the animal model by intravenous injection. They were mainly concentrated in the sinusoidal cells of liver for gene expression (Asayama et al., 1998).

The first gene delivery application of hyaluronic acid was that hyaluronic acid-adipic acid dihydrazide (ADH) hydrogels were used to protect DNA from enzyme degradation and for sustained release of DNA (Shoham et al., 2013). In addition, Yun et al. also prepared hyaluronic acid microparticles colloid in a similar way, and the DNA was incorporated into the gel network for delivery (Yun et al., 2004). These methods of gene delivery using hydrogel have been widely used, especially in tissue engineering. Hyaluronic acid hydrogel was used as warehouse system to control gene delivery in tissue regeneration. In keeping with the same concept, Chun et al. developed a photocross-linked pluronic hydrogel to encapsulate a plasmid DNA (Chun et al., 2005). Hyaluronic acid films and a gene delivery system encoding hyaluronan synthase two have also been developed as the prevention of postoperative peritoneal adhesion membrane barrier membranes with good effect. According to the report, even after the hyaluronic acid membrane was degraded, the release of hyaluronic acid could also be spread by infected neighboring cells to reduce peritoneal adhesion (Kim et al., 2005). Fan et al. first linked the PEI and dexamethasone (Dex), and then made a double-targeted ternary complex hyaluronic acid/PEI-Dex/DNA having a nucleo-shell structure with hyaluronic acid and DNA. The ternary complex showed low toxicity and high transfection efficiency in the tumor cells B16–F10. The intracellular localization showed that hyaluronic acid/PEI-Dex/DNA could promote cellular uptake and DNA nuclear translocation. *In vivo* experiments show that the hyaluronic acid/PEI-Dex/DNA ternary complex had obvious anti-inflammatory activity and tumor growth inhibition in tumor-bearing mice (Fan et al., 2013). Therefore, as a nonviral vector of gene drugs, hyaluronic acid could be targeted to tumor cells through CD44 receptor-mediated endocytosis, and better play the antitumor effect of gene drugs.

Lee et al. reported that hyaluronic acid gel systems delivered siRNA (Lee et al., 2007). The cross-linking of hyaluronic acid could be formed by disulfide bonds, which could be designed to be degraded by glutathione in the cytoplasm. The efficiency of cell absorption and gene silencing was much higher in the CD44 overexpressed cell lines than in the cell lines with lower CD44. The HAP conjugate was also developed to deliver siRNA through LYVE-1-mediated targeting cells (Jang et al., 2014). The external hyaluronic acid in the siRNA/PEI-HA complex was believed to bind to LYVE-1 on the cell membrane of B16F1 (mouse melanoma cells), and then was taken up by the cells via receptor-mediated endocytosis. Based on the distribution of fluorescently labeled siRNA/PEI-hyaluronic acid complexes throughout the body after tail injection, it was shown to accumulate predominantly in the liver and tumor tissues. In addition, when anti-VEGF (endothelial growth factor) siRNA/PEI-hyaluronic acid complexes were intratumorally injected, they effectively inhibited tumor growth to result in the decrease in VEGF product (Park et al., 2010). Hyaluronic acid-spermine polymer could effectively bind to siRNA, self-assemble into micelles (siRNA/HHSCs), and protect siRNA from nuclease degradation. The complex was preferentially taken up by cytoplasmic membrane microcapsule-mediated endocytosis, avoiding degradation of the gene vector by lysosome (Shen et al., 2011). Park et al. used low molecular weight PEI and diacrylamide cystamine to prepare reduced PEI-SS, which reacted with hyaluronic acid to form PEI-SS-hyaluronic acid, and then formed (siRNA/(PEI-SS)-*g*-hyaluronic acid) complex with siRNA to increase serum stability and promote specific targeted uptake of cells. siRNA/(PEI-SS)-*g*-hyaluronic acid had excellent gene silencing efficiency *in vitro*, and the injection of vascular growth factor siRNA/(PEI-SS)-*g*-hyaluronic acid at the tumor site could lower the levels of mRNA and VEGF, and significantly inhibit tumor growth (Park et al., 2011). All these results showed that hyaluronic acid could improve the stability of vector in serum and the efficiency of gene silencing, which had potential value as a target of intracellular delivery of siRNA.

### 2.2. Long-acting conjugates of peptides and proteins

Recently, bioconjugation technology has been applied to synthesize natural polymer compounds, such as polyethyleneglycol (PEG) and hyaluronic acid, which have been widely applied in the development of biopharmaceuticals with good pharmacokinetics (Choi et al., 2011). It was reported that chemical modification of PEG could reduce renal clearance of protein and peptide drugs, reduce immune response and relieve enzyme degradation *in vivo*, thereby increasing efficacy. However, the negative effect of PEGylation has also been reported. Repeated injection of PEGylated liposomes caused long-term cycle decline by the so-called accelerated blood clearance (Ma et al., 2012). In addition, the PEGylated glucagon-like peptide-1 (GLP-1) was reported to significantly reduce its cAMP activity, and the branched pegylation with molecular weight of 43,000 u could even cause greater loss of biological activity (Lee et al., 2006).

As a good candidate to replace PEG, hyaluronic acid has been studied as a new protein and peptide drug carrier. In contrast to PEGylation, each individual chain of hyaluronic acid can conjugate with various numbers of peptide chain molecules, making it possible for polypeptide drugs to exert multiple effects (Jiang et al., 2012). The biological coupling and chemical modification of hyaluronic acid are mostly realized in aqueous solution by the action of hyaluronic acid carboxylic group.

### 2.3. Sustained release of protein drugs

Hyaluronic acid, which is naturally found in the lungs, protects the pulmonary elastin from inflammation, which has mucoadhesive property. According to the study, hyaluronic acid could be used in the delivery of drugs to the lung and nose, and the particles based on hyaluronic acid could prolong the average retention time of drug in the main absorption point of lung (Gratieri et al., 2010). For example, the experiments of encapsulating recombinant human insulin with hyaluronic acid made it a suitable dry powder for inhalation by spray drying, allowing beagle dog to inhale particles into the lung, monitoring the body’s insulin level and insulin-induced glucose level of beagle dog (Surendrakumar et al., 2003). This release kinetics could be controlled by adding excess zinc ions or hydroxypropyl cellulose. Compared with spray-dried pure insulin microparticles, the insulin-encapsulated hyaluronic acid formulation was found to prolong the average retention time and the final half-life, increasing the average residence time by 9-fold and increasing the dose by 2.5-fold with an increase in zinc ions. Moreover, the half-life time was increased (Chu, 2005). These results demonstrated that it was possible to control the delivery of insulin in lung by using particles based on hyaluronic acid.

In order to prolong the release time of protein drugs, hyaluronic acid hydrogels have been extensively studied as a new warehouse system for encapsulating protein drugs (Hirakura et al., 2010). In many cases, protein drugs were rapidly released within a week because of difficulties encountered in preparing highly cross-linked hyaluronic acid microgel network. Further studies showed that the particle size of protein drug was between 3 and 15 nm. When the protein was not degraded, selective cross-linking of hyaluronic acid hydrogel preparation should be developed to form a mesh with a size between 5 and 25 nm hydrogel, which could sustain the release of protein drug through the one-way diffusion.

An alternative cross-linked hyaluronic acid hydrogel was developed as a sustained release of erythropoietin (EPO), which was made using a different pK_a_ between the hydrazide group of hyaluronic acid-ADH and the amino group of protein drug (Motokawa et al., 2006). One type of thiohyaluronic acid formed by disulfide bond was developed as an injection of hyaluronic acid microhydrogel, which was also developed as a control release vehicle for protein drugs. EPO was loaded by the catalysis of sodium terathionate factor in the preparation of hyaluronic acid-SH hydrogel. When sodium tetrathionate was added to the hyaluronic acid-SH hydrogel for preparation, the gelation time was drastically reduced from 1 day to 30 min. EPO release tests *in vitro* and *in vivo* showed that hyaluronic acid particulate hydrogel would have the potential of being a controlled release system for protein drugs (Hahn et al., 2007).

As can be seen from the first three sections, How to design and prepare diversified and intelligent drug carriers, such as temperature-controlled type, pH-controlled type, or induced type by special physiological changes, is the main research direction of hyaluronic acid and its derivatives as drug carriers in the future.

### 2.4. Tumor targeting delivery systems for hyaluronic acid-drug conjugates

Hyaluronic acid-drug conjugates are prodrugs prepared by covalently bonding small molecule antitumor drugs to hyaluronic acid. These covalent bonds are not easily cracked in the blood, but they break through hydrolysis or enzymolysis after reaching the target and release the drug. Hyaluronic acid-drug conjugates can improve the solubility of drug, change the drug distribution and half-life *in vivo*, increase the accumulation of tumor tissue by enhancing the osmotic retention effect, and better exert the efficacy (Fan et al., 2015).

There are three functional groups of carboxyl, amino, and acetyl amino groups on the main chain of hyaluronic acid that can be modified. Therefore, different antitumor drugs can be chemically bonded to form hyaluronic acid-drug conjugates. Galer et al. synthesized the hyaluronic acid-paclitaxel conjugate (HA-PTX), in order to reduce the toxicity of taxanes and improve the antitumor activity (Galer et al., 2011). Hyaluronic acid-paclitaxel had a growth-inhibiting effect on head and neck squamous cell carcinoma cell lines OSC-19 and HN5, and increased the uptake of tumor cells mediated by CD44 receptor. *In vivo* xenograft nude mice pharmacodynamic experiments showed that hyaluronic acid-paclitaxel increased the survival rate of mice, significantly reduced the density of microvessels in tumor tissues, and effectively inhibited the growth of tumors. At the same time, hyaluronic acid-PTX was more effective than paclitaxel free drugs in treating intra-abdominal tumor density, eliminating ascites and prolonging survival time in transplanted ovarian cancer cell lines (Lee et al., 2012b) .

Cisplatin is able to treat most solid tumors, but serious side effects limit its use. Cai et al. made a new type of delivery system linking cisplatin and hyaluronic acid to increase the concentration of platinum in lymphatic vessels, reduce the systemic toxicity and side effects while inhibiting early tumor metastasis (Liu et al., 2015). Compared with the free drug, hyaluronic acid-cisplatin could increase the concentration of drug in plasma and tissue to improve the distribution of drug at the tumor site, greatly reducing the renal toxicity. Hyaluronic acid-sodium butyrate (HA-But) was a tumor cell growth inhibitor sodium butyrate and hyaluronic acid polymer made by chemical bonding. The pharmacokinetics and antitumor activity tests showed that hyaluronic acid-But was accumulated in the liver and spleen after intravenous, abdominal, and subcutaneous administration. Hyaluronic acid-But successfully inhibited hepatic tumor metastasis in Lewis lung carcinoma (LL3) and melanoma (B16–F10) transplanted mouse models. Studies showed that the route of administration had no effect on antitumor activity, but the level of CD44 receptor on the cell surface was related to tumor inhibition rate. The expression level of CD44 on B16–F10 cells was higher than that of LL3 cells. Therefore, the former had stronger antitumor effect (Coradini et al., 2004).

**Figure 1.**
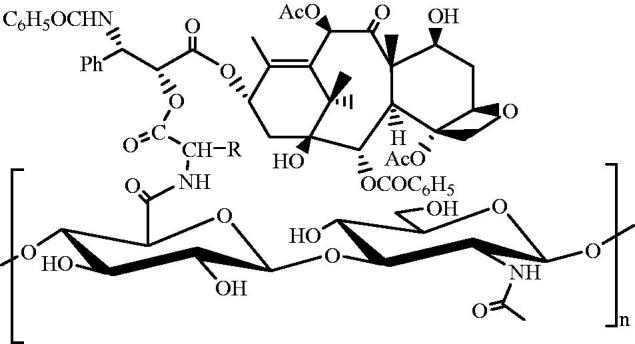
Structure of hyaluronic acid-amino acid-paclitaxel conjugate.

In addition to the direct link with the active groups on hyaluronic acid, small molecular antitumor drugs can be used to prepare hyaluronic acid-drug conjugates by other methods. For example, Xin et al. used amino acid as a cross-linker to link paclitaxel to hyaluronic acid (Xin et al., 2010). First, the carboxyl group of amino acid was linked to the hydroxyl group of paclitaxel, and then the amino group of amino acid was linked to the carboxyl group of hyaluronic acid to make a hyaluronic acid-amino acid-paclitaxel conjugate (Figure 1). In aqueous solution, the amphiphilic conjugate self-assembled into nanoparticles, and paclitaxel was surrounded by a hydrophilic hyaluronic acid shell. Since the presence of amino acids promoted the identification of esterase, paclitaxel was released faster. Cell experiments showed that hyaluronic acid-amino acid-paclitaxel could enhance the toxicity of breast cancer cells, leaving the cell cycle in G(2)/M stage. Therefore, different cross-linking agents could better regulate the drug release rate and provide more options for the preparation of hyaluronic acid-drug conjugate.

### 2.5. The tumor targeting drug delivery system of hyaluronic acid amphiphilic derivatives

Amphiphilic hyaluronic acid derivatives can self-assemble into nanoparticles with core-shell structure in aqueous solution. The internal hydrophobic core of nanoparticles can contain antitumor drugs and contrast agents for diagnosis and treatment (Mayol et al., 2014). The hydrophilic shell prevents unnecessary protein adsorption, thus avoiding the nonspecific uptake of endothelial reticular system. Amphiphilic hyaluronic acid derivatives are used to bind hydrophobic parts (such as lipid soluble molecules, oligomers, and polymers) to the active group of hyaluronic acid. The particle size and zeta potential of self-assembled nanoparticles are controlled by different substitution degrees of the hydrophobic moiety, and generally the proportion of hydrophobic moiety increases, resulting in enhanced hydrophobicity of the inner core and decreased particle size of the nanoparticles.

For the first time, Kim’s team synthesized the hyaluronic acid-ceramide (HA-CE) polymer, and the self-assembled nanoparticles were prepared with Pluronic 85. The nanoparticles could increase cellular uptake, inhibit multidrug resistance, and alter drug release. Due to its short half-life and easy accumulation in the liver, the HA-PE carrier was linked to PEG, which increased the circulating time of HA-CE in the blood, reduced the clearance and increased the accumulation of drug in tumor cells (Cho et al., 2011). Jeong et al. synthesized HA-PLGA block copolymer as a targeting vector for the antitumor drug DOX. The antitumor results showed that the nanoparticles could target human colon cancer HCT-116 cells overexpressing CD44 receptor and enhance cell uptake (Ko et al., 2007).

Lee et al. synthesized amphiphilic HA-5β cholic acid (HA-CA) polymer as an antitumor drug carrier, which effectively increased the uptake of tumor cells, and small particle size nanoparticles were more easily accumulated in tumor tissues than large particles (Lee et al., 2012a) . They used the near infrared dye Cy5.5 to mark HA-CA, and studied the characteristics of carrier in the tumor-bearing mice by noninvasive near infrared fluorescence imaging technology. The results showed that the nanoparticles had a strong fluorescence signal at the tumor site, but a strong fluorescence signal was also found in the liver tissue. This might be due to the uptake of HA-CA nanoparticles by phagocytes of the reticulo-endothelial system (RES). This nonspecific uptake severely restricted the application of hyaluronic acid as an antitumor drug carrier. Therefore, the group used PEG to modify hyaluronic acid nanoparticles, and compared with HA-CE nanoparticles, PEG-modified nanoparticles reduced the uptake of liver and increased the accumulation of tumor sites.

### 2.6. Tumor targeting drug delivery system with hyaluronic acid surface modification

Hyaluronic acid surface-modified nano drug delivery system can not only improve the targeting of nano-preparation, but also relatively extend the circulation time *in vivo*. For example, some liposomes linked with hyaluronic acid, as the targeted part, can enhance the targeting ability of cancer cells and have a higher therapeutic effect (El et al., 2015). Peer et al. prepared the DOX-loaded liposome tHA-LIP modified with hyaluronic acid. Compared with other control groups, the circulation time was longer, the concentration of drug in tumor tissue was higher, the therapeutic effect was better and the systemic toxicity was lower (Peer & Margalit, 2004). Eliaz et al. prepared hyaluronic acid-modified liposomes (HALs) by adding different molar ratios of hyaluronic acid-phospholipid derivatives (HA-PD) to the phospholipid bilayer. HALs was easier to bind to a melanoma cell B16–F10 with a high expression of CD44 receptor, while the binding of fibroblast CV-1 on CD44 receptor negative expression was less. The IC_50_ values of antitumor drug loaded on HALs were significantly lower than those of free drug and unmodified liposomes. In addition, lower drug concentration in HALs showed good antitumor effect (Eliaz & Szoka, 2001).

The hyaluronic acid-modified polylactic acid-glycolic acid copolymer nanoparticles (HCDs) were prepared to increase drug uptake in breast cancer cells. The drug showed sustained release *in vitro* and released 80% in 14 days. Cytotoxicity experiments proved that HCDs had high affinity for cancer cells, and performed more effective antitumor effect (Park et al., 2011a). Rivkin et al. first mixed paclitaxel with lipid, and then linked it with hyaluronic acid to make nanoparticles (PTX-GAGs) (Rivkin et al., 2010). These nanoparticles depended on CD44 receptors to selectively enter tumor cells. When the drug was given to tumor-bearing mice, PTX-GAGs were found to have high safety and could better prevent the growth of tumor.

Superparamagnetic iron oxide nanoparticles coated with hyaluronic acid can increase cancer cell uptake and endocytosis (Thomas et al., 2015). After carrying DOX, it had a killing effect on normal cancer cells and drug-resistant cells. Schneider et al. used hyaluronic acid and cation modified hyaluronic acid to prepare multilayer coating films, and the thickness of the coating was controlled at the nanometer level (Cado et al., 2013). This method could be used to modify the surface of nanoparticles or to study the interaction between the cell and the substrate. The coating film formed by chemical cross-linking of hyaluronic acid and L-lysine played an important role in cell adhesion (Prokopović et al., 2015).

As can be seen from the last three parts, hyaluronic acid has many advantages, such as good biocompatibility, diversity of chemical modification, and targeting of tumor cells. It attracts much attention in the antitumor drug delivery system and provides a good delivery platform for the delivery of oncology therapeutic drugs, which has a great development potential and unique advantage. Hyaluronic acid as antitumor drug carrier research has made great progress, but some problems still need for further study. For example, the use of hyaluronic acid-drug conjugates is limited due to the lack of flexible synthesis methods. Excessive drug or hydrophobic part is bound to the hyaluronic acid main chain, which leads to the change of hyaluronic acid property and affects the receptor-mediated endocytosis process of tumor cells. Therefore, the substitution degree of hyaluronic acid carrier can not only ensure the affinity of hyaluronic acid and receptor, but also inhibit tumor growth to the greatest extent. In addition, due to the presence of hyaluronic acid receptors in hepatic endothelial cells, the drug delivery vector based on hyaluronic acid is accumulated in liver tissues. Although surface modification by PEG can reduce hepatic uptake and increase tumor cell targeting, the density of PEG affects the binding of hyaluronic acid to the receptor. Therefore, it is necessary to study the new type of PEG hyaluronic acid nanoparticles to protect the hyaluronic acid in the circulation process, and to expose the nanoparticles before the uptake of tumor cells for improving the therapeutic effect. When hyaluronic acid is used as an antitumor drug delivery carrier to target tumor cells, the site of action is mainly CD44 receptor (Yu et al., 2013). CD44 receptor may exist a wide range of expression, the mutation itself reduces the selectivity of the target, the update cycle is short, and it is easily saturated. Therefore, it is the future research direction to overcome these shortcomings of CD44 receptor and improve tumor active targeting.

## 3. Conclusion and future perspective

Hyaluronic acid, as a biodegradable polymer, has been used extensively in the controlled-release and targeted drug delivery systems. However, most studies are still only *in vitro* experimental stage, the reports on *in vivo* experiments are rare. However, it is believed that the prospect of hyaluronic acid as drug carriers will be even broader with the discovery of new materials and the development of new technologies. Moreover, the development and utilization of drug carriers with the use of diversity and targeting of chemical modification of hyaluronic acid is still limited. Research on the modification of its derivatives should be strengthened so that it is continuously optimized in biopharmaceutical delivery. In addition, there are a lot of scientific researches on hyaluronic acid as carriers of various drugs at present, but most of them are in the stage of laboratory research. Due to the complex process, hyaluronic acid is difficult to be industrialized. Alchemia in Australia was at the forefront of product development, which was based on hyaluronic acid chemotransport technology (HyACT). The products such as hyaluronic acid-irinotecan, hyaluronic acid-DOX, and hyaluronic acid-5FU were developed at this company. The hyaluronic acid-irinotecan infusion achieved satisfactory results for the targeted therapy of metastatic colorectal cancer in stage I and II clinical trials, and the phase III clinical trials were conducted, but did not achieve the expected results, the reason was not yet clear. Therefore, the industrialization and extensive clinical application of hyaluronic acid as drug carriers is still a long way to go.
